# Achieving universal health coverage with disability inclusion: a household survey analyzing healthcare access and national health insurance utilization for individuals with disabilities in Indonesia

**DOI:** 10.1186/s12913-026-14572-5

**Published:** 2026-04-16

**Authors:** Relmbuss Biljers Fanda, Tri Muhartini, Ika Septiana Eryani, Shita Listyadewi, Ardhina Nugrahaeni, Muhamad Faozi Kurniawan, Retna Siwi Padmawati

**Affiliations:** 1 Center for Health Policy and Management, Universitas Gadjah Mada, Yogyakarta, Indonesia; 2 Erasmus School of Health Policy and Management, Erasmus University, Rotterdam, The Netherlands; 3 Doctoral Program, Faculty of Medicine, Public Health and Nursing, Universitas Gadjah Mada, Yogyakarta, Indonesia; 4 Department of Health Behavior, Environment and Social Medicine, Universitas Gadjah Mada, Yogyakarta, Indonesia; 5 Bioethics Graduate Study Program, Graduate School, Universitas Gadjah Mada, Yogyakarta, Indonesia

**Keywords:** Disability, Access to healthcare, Universal health coverage, National health insurance, Healthcare-seeking behaviors, Out-of-pocket payments

## Abstract

**Background:**

Indonesia, the largest archipelagic nation, is committed to ensuring that persons with disabilities (PWDs) have access to healthcare services without catastrophic costs. This study investigates health-seeking behaviors among individuals with physical and sensory disabilities in Indonesia and the factors associated with their preference for accessing health facilities, utilizing the national health insurance (the JKN) to cover the costs of healthcare services.

**Methods:**

This study employed a cross-sectional design. In 2024, we surveyed 2,666 PWDs across six districts, who were from Yogyakarta City, Bantul, Denpasar City, Buleleng, Kupang City, and Kupang Rural districts, representing variations in access to healthcare, fiscal capacity and healthcare system developments. The outcomes of this study encompassed PWDs’ healthcare-seeking behaviors, their JKN utilization and out-of-pocket payments.

**Results:**

Most PWDs in these districts reported a high need for health services; however, only 50% of them accessed health facilities. Although a high rate of insurance enrollment around 88%, was observed, barriers to accessibility and financial capacity for accessing healthcare service points still persist. Regression analyses revealed that having diagnoses made by healthcare workers was associated with the outcomes, whereas being registered with the JKN was associated with seeking assistance. Health service costs for those accessing primary healthcare centers and private clinics were affordable, whereas the costs at the hospital level were not. Population access and the JKN utilization differed across districts.

**Conclusions:**

Although Indonesia is making progress toward universal health coverage, by successfully enrolling almost PWDs and ensuring affordable health services in primary care, low rates of access to health facilities and use of the insurance still persist. Tackling those challenges requires not only health-sector interventions but also socio-economic programs. Enhancing local government efforts to overcome district-specific barriers and variations in progress can speed up the integration of a disability inclusion agenda.

**Supplementary Information:**

The online version contains supplementary material available at 10.1186/s12913-026-14572-5.

## Background

Improving access to healthcare for persons with disabilities (PWDs) is a crucial component of achieving universal health coverage (UHC) [1, 2]. Globally, approximately 1.3 billion individuals live with disabilities [3]. This population frequently experiences greater healthcare needs and worse health outcomes, placing them among the most vulnerable groups. Barriers to accessing care, including stigma, discrimination, and the high cost and complexity of treatment, are well-documented, particularly among those with limited income and education [4].

While advancing its UHC agenda, Indonesia, a country with a population of more than 270 million people, must ensure that approximately 28 million individuals with disabilities, representing one in every ten Indonesians, are not left behind [5, 6]. In 2014, a bold political agenda to register all Indonesians in its national health insurance (the JKN) scheme was started and has achieved almost full participant coverage (99%) by the beginning of 2025 [7, 8]. For individuals with disabilities living with limited financial resources—who make up four in ten of all PWDs—the government fully covered their JKN premiums [9]. They must be registered in the Integrated Social Welfare Database to qualify for this benefit. Evidence for the country to enhance and accelerate its health efforts to safeguard the needs of this vulnerable population is needed.

Striving to achieve full Indonesian UHC leads to a vision of ensuring access to healthcare services without catastrophic payments for all citizens, including PWDs [10]. A national commitment was demonstrated through the enactment of a new health law in 2023, reaffirming its commitment to improving access to healthcare for individuals with disabilities [11]. All health services should be free of charge when accessing primary healthcare centers (PHCs), hospitals, private clinics, and independent general practitioners or midwife practices that collaborate with the national health insurance agency, BPJS Kesehatan [12]. This applies only when patients follow the JKN referral system, in which care starts at a primary healthcare center or private clinic and is referred to a hospital if needed. As it is designed and written in the concept of the JKN and its complementary program for social assistance, individuals with disabilities should not encounter any challenges in paying their JKN premiums and health services [13].

Until 2025, Prior surveys conducted indicated that individuals with disabilities are struggling to access health services, showing that the progress of the JKN implementation is still a long way from its vision. A study conducted by the Indonesia Corruption Watch (ICW) in four cities (Bandung, Solo, Makassar, and Kupang) revealed that 72% of 800 PWDs were aware that they were already registered in the national insurance scheme. It also noted that 95% of them were unwell and had already sought health services when needed [14]. Another survey from the Indonesian statistics agency revealed that only 41% of 28 million PWDs accessed health services when needed, and 32% of them stated using the JKN to cover their health services [6]. The results of this study were contrasted by another study that identified the JKN as the primary source of funds for the healthcare access of PWDS [15]. More concerning, analysis of a national socioeconomic dataset showed that individuals with disabilities have higher healthcare utilization rates than the general population, but also incur greater out-of-pocket (OOP) expenses and face an increased risk of catastrophic health expenditure [16].

The diverse setting of the largest archipelagic country globally poses challenges for the general population and will be worsened for those with disabilities in accessing healthcare facilities. Indonesia has more than 17,000 islands; however, one-third of its population resides on the islands of Java and Bali, constituting 6% of the landmass. The remainder of the population is scattered across the other 8,000 islands, causing an uneven population density throughout the country [10]. This variation is also somehow connected with poverty rates, as a province in Papua has ten-fold more of its population living below the poverty line than a province in Java. A similar situation exists in the health sector developments. Individuals living in Java and Bali have significantly easier access to hospitals with medical subspecialties than those residing in rural areas outside the region [17]. More concerning, some of this disadvantaged population have no hospitals in their districts and have limited income [18]. While each subdistrict in Indonesia is equipped with one or two PHCs, the provisions of general practitioners and medications are frequently constrained in the less affluent districts [17, 19, 20]. These structural and geographic barriers have long constrained access to quality healthcare for the broader population and remain persistent systemic challenges. For persons with disabilities, accessing care often requires additional support, yet they continue to face compounded barriers, including higher out-of-pocket costs for transportation, accommodation, and caregiver assistance. These needs are not fully addressed by JKN and other national health and social programs, raising concerns about the extent to which PWDs benefit from JKN’s promise of financial protection in accessing healthcare services [12, 21].

Furthermore, another opportunity or challenge emerges from the multilayer healthcare system structure, which emphasizes improving the performance of sub-national health systems in advancing the disability inclusion agenda. As decentralization began in 2001, the district governments have had their own authority and responsibility to decide and develop their local programs, including public health. There are 514 district health offices operating according to their local contexts [22]. Districts, including those located in Java and Bali, generally possess greater fiscal capacity than those situated in other areas, making them a better choice for developing health programs. For example, the government of Yogyakarta offers a local fund for medical device technology for individuals with disabilities and other direct medical costs not covered by the JKN scheme. Nevertheless, not all districts in Indonesia have prioritized improving access to healthcare for persons with disabilities. This is because the local government may consider other health issues, such as stunting, malaria, and maternal mortality, to be more urgent or due to limited funds, even though it has been mandated to incorporate the disability agenda into its local health development programs.

Understanding the knowledge gaps in health-seeking behaviors among PWDs in Indonesia, as well as the factors influencing their decisions to seek healthcare, is crucial for assisting the Indonesian government at national and sub-national levels to better design health initiatives for disability inclusion. However, evidence regarding healthcare access for PWDs, starting with healthcare need identification, followed by seeking services, reaching resources, and using the necessary services, remains limited [23]. These assessments regarding current access at the national and district levels are crucial for evaluating the effectiveness of these initiatives and addressing their future goals.

This study aimed to fill the gap by identifying the determinants of health-seeking behaviors among individuals with disabilities and their utilization of the JKN to protect themselves from financial hardship. We conducted a household survey of 2,666 households with individuals who have physical or sensory disabilities. Data were collected across six districts, evenly divided between urban districts (cities) and rural districts in three provinces. Individual characteristics, including sex, education level, income, and the availability of a guardian, are also included in our analyses. This paper examines how PWDs use JKN for general health needs, while their experiences with accessing disability-specific benefit packages, such as occupational services, will be covered in a separate work. Our analysis draws on the health-seeking behavior theory for assessing the accessibility and affordability of healthcare services [24–26]. We focus on the dimensions of financial protection and population coverage, although health services covered by the JKN are not part of our analyses [27]. Regarding those who do not utilize national insurance, we also investigated how much they spend out of pocket when seeking care.

## Methods

### Study design and setting

This paper is part of our research project Strengthening and Accelerating Marginalized Groups’ Access to Healthcare and Knowledge (SAMA-HAK), which aims to analyze the health service benefits packages under Indonesian Universal Health Coverage (UHC), known as JKN, with a focus on people with physical disabilities. This program was funded by DFAT Australia to assist the Government of Indonesia in using evidence within their policy-making process and to enable its existing Indonesian Civil Society Organizations, known as INKLUSI partners, to use evidence for their advocacy agenda in strengthening disability agenda in Indonesia.

This study employed a cross-sectional design with a randomized sample and was conducted in the Special Region of Yogyakarta (Bantul and Yogyakarta City), Bali (Buleleng and Denpasar), and East Nusa Tenggara – NTT (Kupang Rural and Kupang City). We purposefully selected three provinces not only for their high prevalence of PWDs but also to represent Indonesia’s geographic and economic diversity. The special region of Yogyakarta provides better access to healthcare, districts in Bali demonstrate high fiscal capacity and better health development and access, whereas districts in NTT exhibit limited income and healthcare development options [12, 17, 28]. These locations also included local networks associated with the INKLUSI partners: Sigab and Yakkum. This study was conducted from June 2023 to April 2024, integrating urban–rural dynamics during the selection process of sites.

### Respondents

This study focused on individuals with physical, sensory, and multiple disabilities, referring to physical and sensory disabilities. Respondent classification was based on Indonesian definitions of disability based on their types [29]. Physical disabilities include mobility impairments, such as paralysis or limb loss. Sensory disabilities denote impairments in one or more senses, including visual, hearing, and speech impairments. Multiple disabilities refer to individuals who experience both physical and sensory impairments.

To ensure participant selection’s accuracy, efficiency, and effectiveness, we applied clear inclusion and exclusion criteria. The following were the inclusion criteria: (1) individuals aged ≥ 18 years, (2) those registered in the Social Services Agency (Dinas Sosial) database at the district/city or provincial level, (3) those who could directly participate in this study or designate a family member to do so, and (4) individuals who had resided in the selected district or city for > 6 months. The following were the exclusion criteria: (1) individuals who were unable to effectively communicate to respond to the research questions and (2) those whom the enumerators could not contact after three attempts.

The sample size was determined on the basis of a total population of approximately 11,800 PWDs, as reported in administrative records from the Social Services Offices in the three provinces, including both those enrolled in the JKN system and those who are not. The final sample size was calculated using the following formula:

$$\begin{gathered} Sample{\text{ }}size \hfill \\ n = {\text{ }}\left[ {{\mathbf{DEFF}}*{\mathbf{Np}}\left( {{\mathbf{1}} - {\mathbf{p}}} \right)} \right]/ \hfill \\ [\left( {{{\mathbf{d}}^{\mathbf{2}}}/{{\mathbf{Z}}^{\mathbf{2}}}_{{\mathbf{1}} - {\mathbf{\alpha }}/{\mathbf{2}}}*\left( {{\mathbf{N}} - {\mathbf{1}}} \right) + {\mathbf{p}}*\left( {{\mathbf{1}} - {\mathbf{p}}} \right)} \right] \hfill \\ \end{gathered} $$

N = population size (for finite population correction factor or fpc)

p = hypothesized % frequency of outcome factor in the population

d = confidence limits as % of 100(absolute +/− %)

*DEFF =* design effect

To anticipate potential dropouts, we added 10% to the calculated total sample size. Consequently, the final number of respondents registered in this study was 2,666 PWDs.

We conducted location stratification for data collection based on urban wards (*kelurahan*) and rural villages (*desa*) within each district and province. A randomization process was applied to select these *kelurahan* and *desa*. If a respondent was registered twice with slight differences in their names; respondents had moved or passed away; or the village head was unaware of the PWDs’ whereabouts, replacements of a participant from the same village and same disability group were made. In a case the village had no further potential respondents from the same group, additional locations were gradually included through randomised selection until the target sample size was reached.

The detailed sample distribution, disaggregated by urban and rural areas in each district, is presented in Table 1.

**Table 1:** Sample sizes in this survey based on locations and types of disabilities

Province	District	Type of disability	Numbers of Population	Our samples
DIY	Yogyakarta City	Vision Impairment	220	44
		Hard of Hearing	288	81
		Physical disability	948	285
		Multiple disabilities	80	55
	Bantul	Vision Impairment	541	50
		Hard of Hearing	622	69
		Physical disability	1830	329
		Multiple disabilities	125	43
Bali	Denpasar City	Vision Impairment	36	32
		Hard of Hearing	826	265
		Physical disability	286	101
		Multiple disabilities	15	13
	Buleleng	Vision Impairment	370	51
		Hard of Hearing	649	71
		Physical disability	3004	353
		Multiple disabilities	76	32
East Nusa Tenggara	Kupang City	Vision Impairment	136	47
		Hard of Hearing	85	57
		Physical disability	314	183
		Multiple disabilities	275	109
	Kupang Rural	Vision Impairment	122	41
		Hard of Hearing	195	54
		Physical disability	655	240
		Multiple disabilities	102	61
		**Total**		2666

### Data management

At the initial stage of this study, we developed and adapted our research questionnaire by referring to several sources, including the *Model Disability Survey* by the World Health Organization and World Bank, the 2023 National Socioeconomic Survey (SUSENAS), and the *Disability Health Service Access and Quality Survey* by the ICW. During the development process, we involved organizations of PWDs, the Ministry of Health, and the INKLUSI program to review and provide feedback on our questionnaire. Following reviewer approval, the finalized questionnaire was digitized using the *KoBoToolbox* platform. Our final instrument is available at 10.5281/zenodo.18095036.

The next step was to conduct a pilot test in the following three selected regions: Kupang City (East Nusa Tenggara), Buleleng District (Bali), and Bantul District (Special Region of Yogyakarta). The pilot study involved 30 respondents to ensure that the questions were clearly understood and appropriately answered by participants. Although we did not extend the pilot test to include validity and reliability assessments to ensure the quality of the survey items, all feedback was thoroughly discussed in our meeting, and revisions were made on the basis of the findings of the pilot test.

We conducted face-to-face surveys directly with PWDs. For those PWDs with hard of hearing, sign language interpreters were hired to facilitate the interviews. Overall, 30 enumerators and 3 field coordinators participated in the data collection process, with each interview session spanning approximately 30–50 min.

To ensure the quality and credibility of the survey data, we applied spot checks and cross-checking mechanisms. Field coordinators accompanied different enumerators on a rotating basis for daily spot checks. Furthermore, the researchers reviewed incoming data daily. Upon reaching 50% of the data collection target, we conducted cross-checks on 10% of the collected data by revisiting the respondents and reperforming the interviews to verify the accuracy of their initial responses. In cases where discrepancies were noted, we used the responses obtained during the follow-up interviews as the final data.

## Variables

### Access to healthcare services

Access to healthcare services has been classified as the act of utilizing one of the following health facilities: PHCs, hospitals, private clinics, and independent general practitioner practices, in accordance with the established classification of health facilities. One respondent can select more than one healthcare facility. As the acquisition of medications from drug stores does not indicate a requisite systematic treatment for diseases, it is not categorized as access to healthcare services. This variable is designated by the following two labels: having ever accessed healthcare, regardless of the number of visits in one or more facilities (Value 1) and not accessing healthcare (Value 0).

### Using the JKN

As several options for accessing healthcare are available, we continue to investigate the decision to use the JKN in each healthcare facility, as reported by the respondents. A value of 0 indicates not using the JKN, indicating that they bear costs independently, whereas a value of 1 signifies full coverage by the JKN (free of charge), as long as the individual follows the structured referral system required by JKN.

### Financial costs of not using the JKN

When respondents indicated that they did not use JKN, we asked how much they paid for medical bills. These data serve as a measure of OOP payments. We observed that 377 respondents who have JKN reported OOP, and 97 samples were not yet registered. Some of the respondents could not remember the health service prices, resulting in missing data. We did not treat any missing data. The costs were compared to the regional minimum daily wage to assess affordability.

### Statistical analyses

**Fig. 1:**
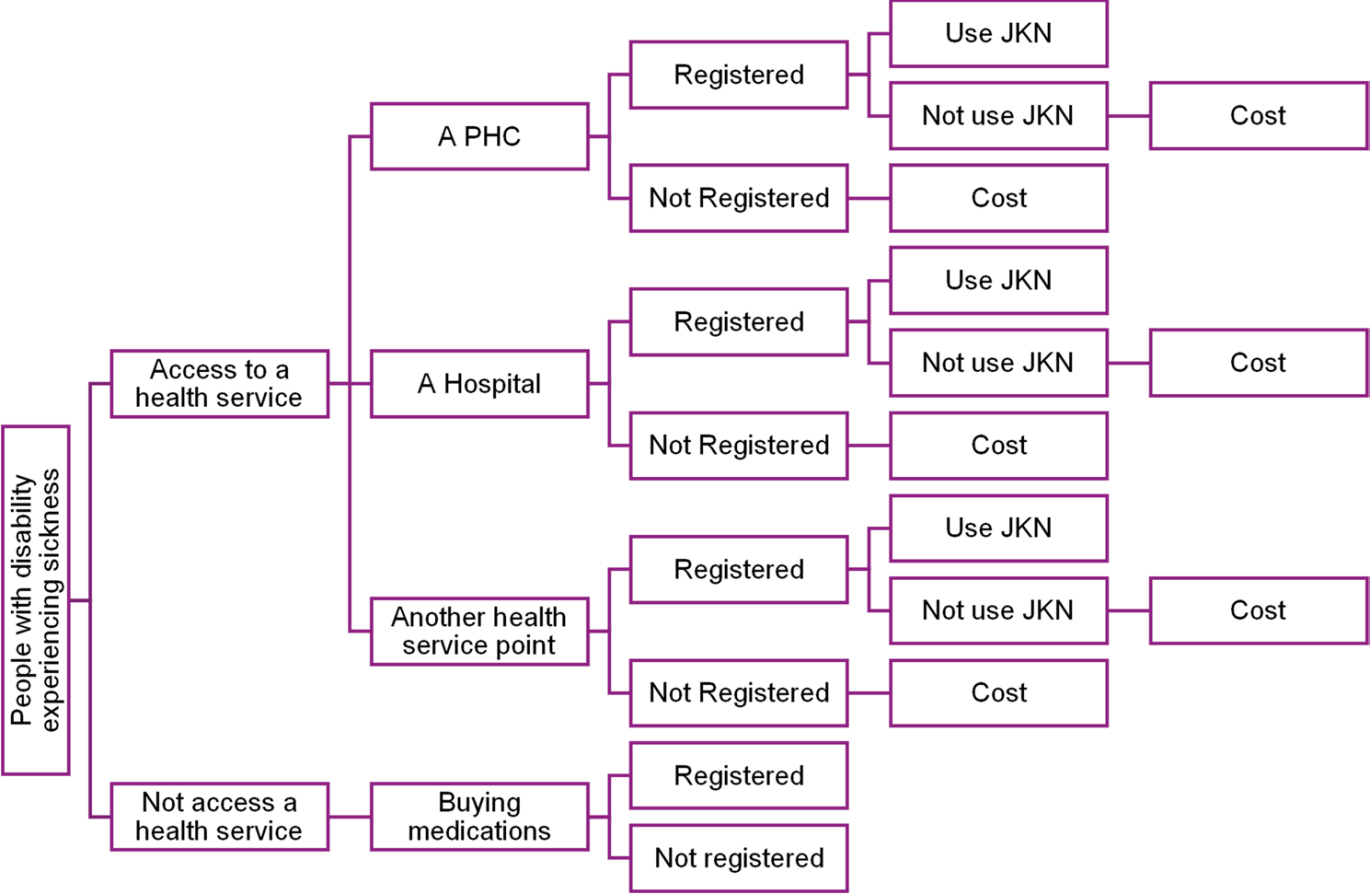
Our logical approach in this study

We conducted a descriptive and bivariate analysis of healthcare access and utilization of the JKN. We calculated the percentage of each variable and examined their associations using chi-square analyses to identify individual relationships. We described the OOP data using the median and interquartile range since they were not normally distributed (normality test results). We performed logistic regressions for the factors associated with the outcomes in the chi-square test. We constructed the regressions on the basis of our logical framework using a patient-centered access to health care framework [23], as illustrated in Fig. 1. We examined the healthcare needs of Persons with Disabilities over the past year, along with their decisions about whether to seek healthcare services. The decision to utilize the required services involves not only where they access these services but also the use of the National Health Insurance (JKN) in their treatment. This paper focuses specifically on healthcare costs and does not explore more detailed aspects of healthcare utilizations, such as whether visits are initial or follow-up, nor do we assess healthcare outcomes.

Data management and analyses were performed using STATA 19 BE (personal license of the first author, RBF), and Microsoft Excel was employed for generating the graphs (institutional license via Erasmus University Rotterdam).

## Results

We here present data regarding the distribution of PWDs based on their national health insurance (JKN) utilization across six districts. A total of 53 respondents were excluded from our analysis due to their reports of never experiencing illness within the past 12 months. Descriptive statistics pertaining to our respondents, emphasizing healthcare accessibility and their choices regarding JKN utilization, is shown in Table 2. We observed variation in JKN enrolment and utilisation across the study locations, as illustrated in Supplementary Fig. 1. The outcomes derived from our logistic regression models for both factors are delineated in Table 3. Concurrently, data related to OOP expenses incurred by PWDs when not utilizing the JKN to cover their bills are illustrated in Table 3. Our findings are presented on the basis of the two outcomes.

**Table 2:** Distribution of respondents based on access to health services and their decision to utilize the JKN for coverage of these services

Characteristics	Access a health facility			*P*-value	Using JKN			*P*-value
	No	Yes	Total		No	Yes	Total	
	1305(49,94%)	1308(50,06%)	2613(100%)		304(23,15%)	1009(76,85%)	1313(100%)	
Registered as a JKN participant	1106(47,65%)	1215(52,35%)	2321(100%)	< 0.001				
*Sex*								
Female	575(50,22%)	570(49,78%)	1145(100%)	0,803	130(22,73%)	442(77,27%)	572(100%)	0.175
Male	730(49,73%)	738(50,27%)	1468(100%)		174(23,48%)	567(76,52%)	741(100%)	
JKN subsidized participant - PBI JKN	976(49,27%)	1005(50,73%)	1981(100%)	0.222	260(24,03%)	822(75,97%)	1082(100%)	0.103
*Age groups*								
Young people (18–24)	138(57,98%)	100(42,02%)	238(100%)	< 0.001	28(28,28%)	71(71,72%)	99(100%)	0.128
Adult (25–44)	410(56,79%)	312(43,21%)	722(100%)		75(25,86%)	215(74,14%)	290(100%)	
Middle-aged people (45–60)	414(48,08%)	447(51,92%)	861(100%)		90(20,13%)	357(79,87%)	447(100%)	
Senior citizens	343(43,31%)	449(56,69%)	792(100%)		111(23,27%)	366(76,73%)	477(100%)	
*Occupation*								
Having a Job	497(49,31%)	511(50,69%)	1008(100%)	0.606	105 (28,87%)	398(79,13%)	503(100%)	0.754
Self-employment	144(49,66%)	146(50,34%)	290(100%)	0.917	46(32,62%)	95(67,38%)	141(100%)	0.001
Work in the formal sector	27(36,49%)	47(63,51%)	74(100%)	0.019	10(22,22%)	35(77,78%)	45(100%)	0.881
Work in the informal sector	338(49,20%)	349(50,80%)	687(100%)	0.650	61(17,43%)	289(82,57%)	350(100%)	0.038
*Level of education*								
* Never attended*	414(58,97%)	288(41,03%)	702(100%)	< 0.001	78(28,06%)	200(71,94%)	278(100%)	0.008
Have ever been a student	854(47,31%)	951(52,69%)	1805(100%)		189(19,65%)	773(80,35%)	962(100%)	
Have ever been a university student	37(34,91%)	69(65,09%)	106(100%)		13(17,81%)	60(82,19%)	73(100%)	
Having a guardian	673(48,95%)	702(51,05%)	1375(100%)	0.283	164(22,94%)	551(77,06%)	715(100%)	0.119
*Type of disability*								
Vision Impairment	144(55,81%)	114(44,19%)	258(100%)	0.047	22(18,03%)	100(81,97%)	122(100%)	0.351
Hard of Hearing	176(41,61%)	247(58,39%)	423(100%)	0.001	53(23,04%)	177(76,96%)	230(100%)	0.484
Physical disability	801(49,32%)	823(50,68%)	1624(100%)	0.417	185(11,46%)	655(40,58%)	1614(100%)	0.410
Multiple disabilities	184(59,74%)	124(40,26%)	308(100%)	0.001	20(16,53%)	101(83,47%)	121(100%)	0.176
Married	490(43,52%)	636(56,48%)	1126(100%)	0.001	139(21,06%)	521(78,94%)	660(100%)	0.814
Using a medical-assisted device	434(49,15%)	449(50,85%)	883(100%)	0.563	87(18,63%)	380(81,37%)	467(100%)	0.076
Diagnosed by a health worker	303(30,89%)	678(69,11%)	981(100%)	< 0.001	121(17,24%)	581(82,76%)	702(100%)	< 0.001
*District Name*								
Yogyakarta City	202(44,69%)	250(55,31%)	452(100%)	0,014	33(12,5%)	231(87,5%)	264(100%)	< 0.001
Bantul	275(57,65%)	202(42,35%)	477(100%)	0,001	63(29,3%)	152(70,7%)	215(100%)	0.002
Denpasar	172(42,05%)	237(57,95%)	409(100%)	< 0.001	64(28,83%)	158(71,17%)	222(100%)	0.003
Buleleng	171(34,76%)	321(65,24%)	492(100%)	< 0.001	108(35,41%)	197(64,59%)	305(100%)	< 0.001
Kupang City	254(64,63%)	139(35,37%)	393(100%)	< 0.001	4(2,84%)	137(97,16%)	141(100%)	< 0.001
Kupang	231(59,23%)	159(40,77%)	390(100%)	< 0.001	8(4,82%)	158(95,18%)	166(100%)	< 0.001
*Types of Districts*								
Urban district	628(50,08%)	626(49,92%)	1254(100%)	0.893	101(16,11%)	526(83,89%)	627(100%)	< 0.001
*Province*								
Special Region of Yogyakarta	477(51,35%)	452(48,65%)	929(100%)	0,287	96(20,04%)	383(79,96%)	976(100%)	0.390
Bali	343(38,07%)	558(61,93%)	901(100%)	< 0.001	172(32,64%)	355(67,36%)	860(100%)	< 0.001
East Nusa Tenggara	485(61,94%)	298(38,06%)	783(100%)	< 0.001	12(3,91%)	295(96,09%)	735(100%)	< 0.001
*Access to healthcare facilities*								
PHC					26(4,22%)	590(95,78%)	616(100%)	< 0.001
Hospital					34(9,83%)	312(90,17%)	346(100%)	< 0.001
Access to other health facilities					220(62,68%)	131(37,32%)	351(100%)	< 0.001

**Table 3:** Final logistic regression models on factors associated with accessing health services and using the JKN

	Odds ratio (95% CI)	Pseudo R2
**Access to a health service**		
Having JKN	2,14(1,62:2,85)***	*11*,*6%*
Level of education		
General school	1,59(1,30:1,94)***	
Higher Education	2,59(1,64:4,10)***	
Hard of Hearing	1,97(1,57:2,49)***	
Marriage	1,20(1,01:1,43)*	
Diagnosed by a health worker	3,42(2,87:4,08)***	
Denpasar	1,57(1,23:2,00)***	
Buleleng	2,46(1,95:3,10)***	
Kupang city	0,68(0,53:0,87)**	
***Using the JKN***		*36*,*4%*
Yogyakarta city	0,28(0,13:0,61)**	
Bantul	0,17(0,08:0,35)***	
Denpasar	0,29(0,14:0,6)***	
Buleleng	0,20(0,10:0,40)***	
Diagnosed by a health worker	1,51(1,07:2,15)*	
PHC	3,02(1,90:4,82)***	
Hospital	1,20(7,67:1,89)***	

* p-value <,*05*

** p-value <,01

*** p-value <,001

### The JKN enrolment coverage

Our survey results showed that 89% of respondents were enrolled in this social health insurance scheme, showing a high level of insurance coverage. There was no substantial difference between those living in cities and regencies. However, differences emerged at the district level: coverage in two districts in Yogyakarta Special Region (DIY) was higher compared to four districts in the other two provinces. Yogyakarta City, in particular, is approaching full coverage, with 96% of PWDs enrolled in JKN. In contrast, enrolment in Kupang Regency was only 83%, where health service facilities are more limited, and residents have a lower capacity to pay for care.

Registration rates varied between two districts in the same province, as district-level health systems organise their health system, social programs and disability inclusion agendas differently. For example, in the Special Region of Yogyakarta, nearly half of PWDs in Yogyakarta City—the highest rate—used JKN, compared with only a third in neighbouring Bantul.

### Access to healthcare services

**Fig. 2:**
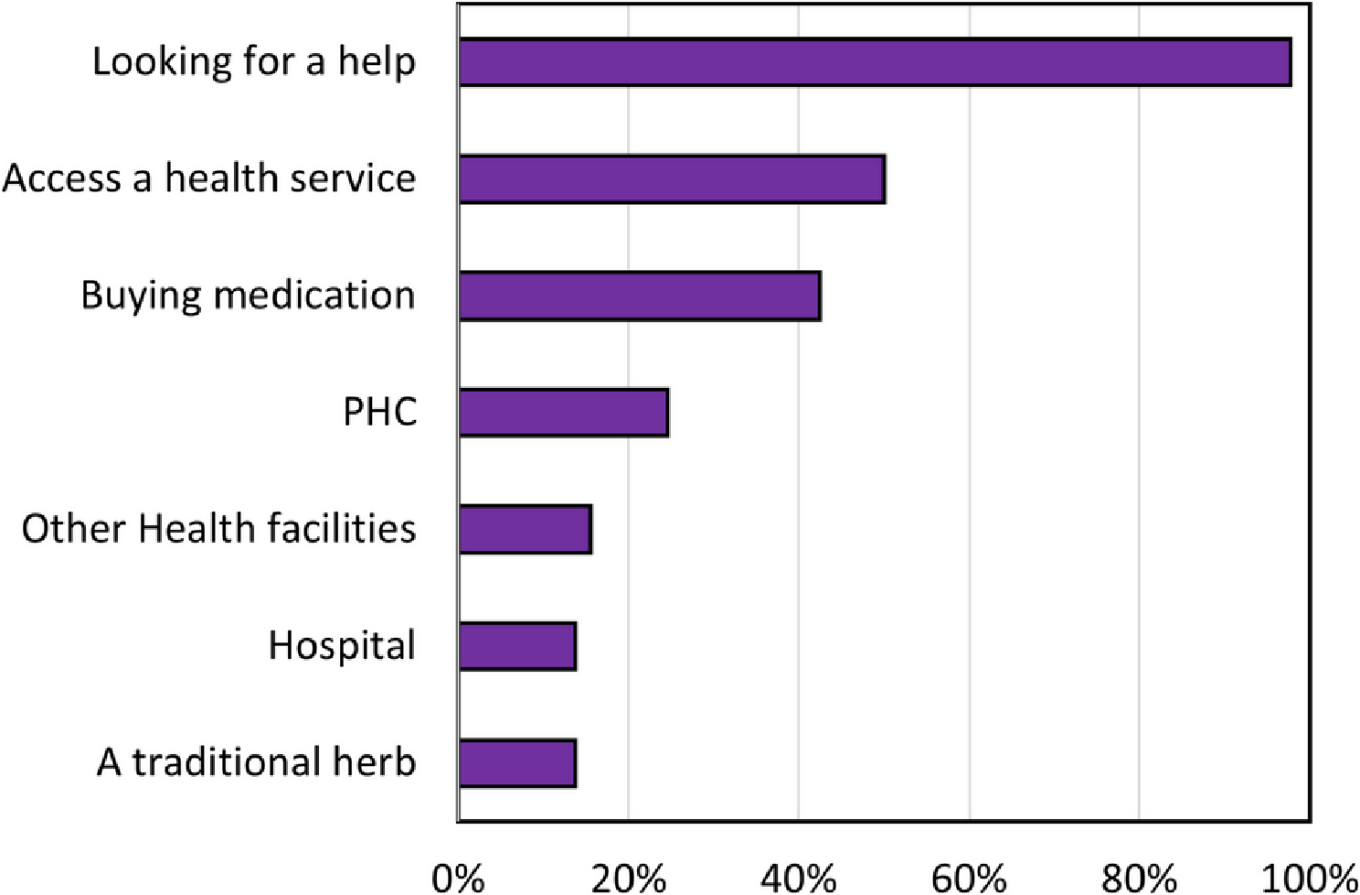
Distributions of people with disabilities’ health-seeking behaviour (N:2613)

Of the 2,666 respondents, almost all PWDs indicated that they need healthcare services (98%), and nearly all of them were looking for help (98%) (Fig. 2). Of the respondents, only 50% respondents decided to seek a health service in a health facility, while 43% bought medicines without consulting a healthcare worker. Only six PWDs decided to do nothing when they needed help, which is considered very small.

Over 40% of PWDs reported visiting a PHC (puskesmas), with even fewer opting for other health facilities or hospitals. This finding occurs because PHCs are the most accessible, usually with one or two per district. Going directly to hospitals without first visiting a PHC or obtaining a referral letter from a PHC results in the JKN not covering their medical bills.

**Fig. 3:**
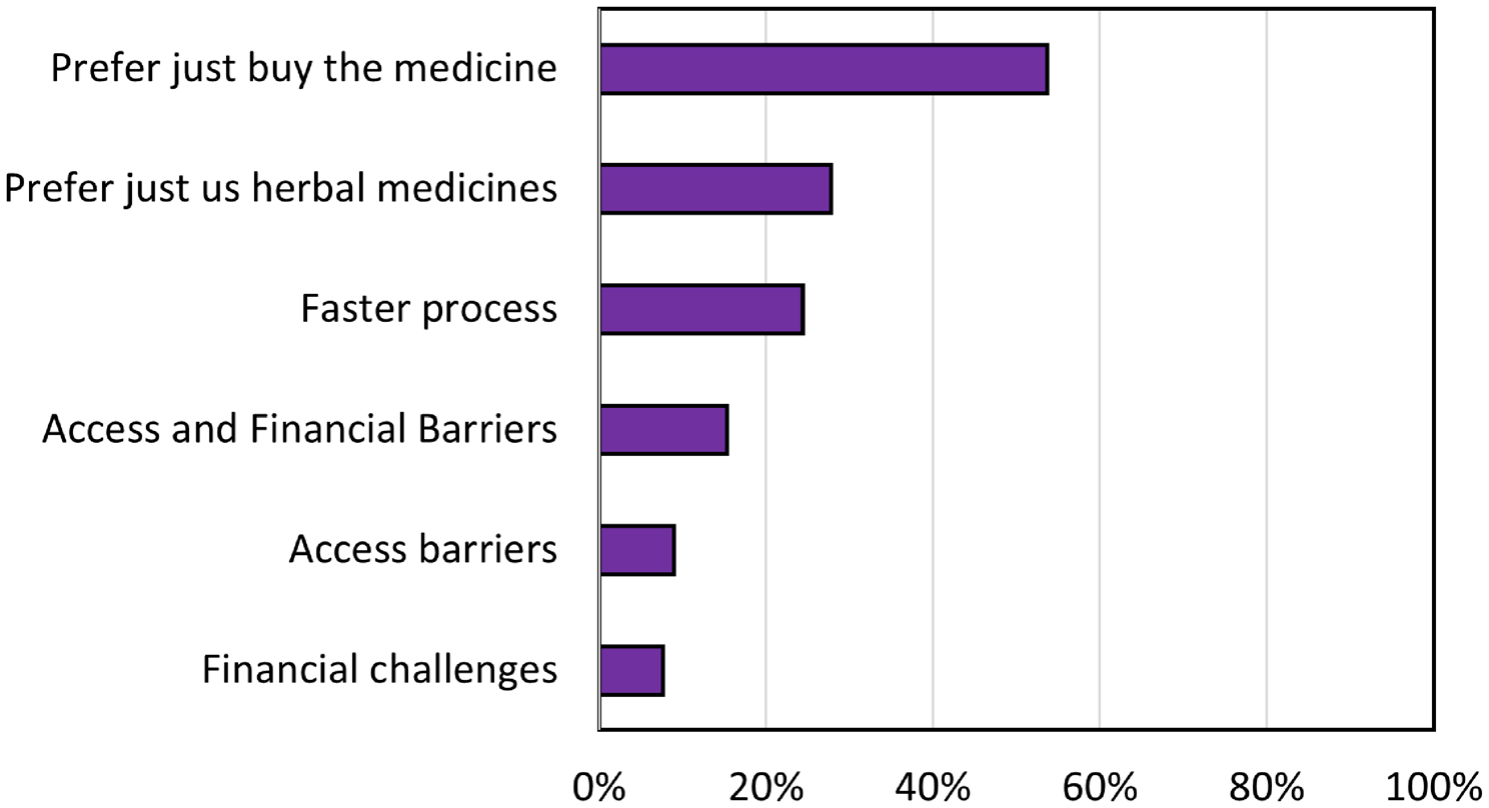
Distributions of reasons for people with physical and sensory disabilities to no access healthcare services six districts in Indonesia (*N* = 1292)

Further analysis of the reasons why 1,292 PWDs are not accessing legitimate health services provided by the BPJS Kesehatan is presented in Fig. 3. They exclusively preferred to purchase medication without any prescription by healthcare workers – self-medication, revealing that > 54% of this population already knows which medication they need, whereas 25% opted for traditional treatments. However, 10% of individuals in this population reported experiencing difficulties in accessing healthcare services owing to excessive treatment costs or geographic barriers, and no respondents expressed concern about the poor quality of health services.

Our logistic regression in Table 3 reveals that those diagnosed by a healthcare worker are most likely to access healthcare services (odds ratio [OR], 3.42; 95% confidence interval [CI], 2.87–4.08). However, only 981 respondents (38%) reported being diagnosed by a healthcare worker from prior healthcare utilization. Registering as a JKN participant, having a better education, and being hard of hearing were the three factors strongly associated with the outcome. Moreover, married individuals were 20% more likely to access healthcare when they were sick. Those living in Denpasar and Buleleng districts were also more likely to access healthcare.

### Utilize the JKN 

Although insurance enrolment is high, it does not necessarily translate into effective coverage that improves healthcare access for PWDs. Our findings indicated those 40% of the respondents were enrolled in the JKN and utilized healthcare services at PHCs, hospitals, or other healthcare facilities (private clinics and independent healthcare worker practices). Similar usage rates were observed across provinces and districts observed. Within this cross sectional design, 96 individuals accessed multiple types of facilities, whereas 2 individuals availed themselves of all three facilities.

By restructuring the datasets on healthcare facility service points, we identified 1,313 decisions pertaining to payment patterns across the three types of healthcare facilities. Approximately half of these decisions (47%) were derived from PHCs, whereas the remainder was evenly distributed between hospitals and other services.

Most of these decisions involved JKN utilization to cover treatment costs. Furthermore, we noted that in both the urban district of Kupang and the rural districts, almost all decisions (> 95%) were associated with JKN utilization. Consequently, owing to the lack of variation, this variable could not be included in the logistic regression model.

Individuals visiting PHCs demonstrated a statistically significantly higher likelihood of using the national health insurance scheme (JKN) for healthcare coverage (OR, 3.02; 95% CI, 1.90–4.82; Table 3). Additionally, those who received a diagnosis from a healthcare professional had a slightly better likelihood of utilizing JKN services (OR, 1.5; 95% CI, 1.07–2.15). Furthermore, hospital visit was correlated with increased JKN utilization, emphasizing the reliance on this national scheme. In contrast, respondents living in Yogyakarta City, Bantul, Denpasar, and Buleleng were significantly less likely to use JKN than those residing in Kupang City and Kupang District.

### Financial costs of not using the JKN

We noted 327 instances of OOP expenses, which we descriptively analyzed in relation to JKN ownership, study location, and the healthcare facilities visited. By comparing OOP expenses to the regional minimum daily wage in a 1:1 ratio as a measure of affordability, our findings indicated that costs were generally manageable at PHCs and other healthcare facilities. In contrast, hospital expenses were considered unaffordable, especially for those without JKN coverage. This difference in affordability was evident across various study regions.

**Table 4:** Distributions of out-of-pocket payments for individuals with disabilities across six districts in Indonesia, analysed by health facilities

Districts	PHC		Hospital		Other		Total	
	*N*	Median (IQR)	*N*	Median (IQR)	*N*	Median (IQR)	*N*	Median (IQR)
Register as the JKN participants								
Yogyakarta City	6	0,30(2,63)	16	1,89(2,09)	17	0,95(0,76)	39	1,42(2,18)
Bantul	3	1,06(3,86)	14	2,13(6,71)	56	0,53(0,21)	73	0,53(0,75)
Denpasar City	2	0,61(0,99)	4	2,75(1,03)	44	0,73(0,57)	50	0,73(0,92)
Buleleng	10	0,45(0,40)	0	0,00(0,00)	72	0,57(0,16)	82	0,57(0,16)
Kupang City	4	0,05(0,08)	3	3,52(3,92)	0	0,00(0,00)	7	0,10(3,47)
Kupang Rural	4	0,17(1,40)	3	1,91(2,97)	7	1,04(0,52)	14	1,04(1,55)
Sub-total	29	0,34(0,58)	40	2,13(4,68)	196	0,61(0,34)	265	0,65(0,76)
Not registered as JKN participants								
Yogyakarta City	4	0,09(0,09)	2	4,26(4,73)	2	1,09(1,61)	8	0,25(1,81)
Bantul	0	0,00(0,00)	1	4,26(0,00)	1	0,53(0,00)	2	2,40(3,73)
Denpasar City	1	1,47(0,00)	2	5,23(4,22)	14	0,73(0,88)	17	1,10(2,31)
Buleleng	2	0,32(0,16)	1	3,64(0,00)	19	0,57(0,32)	22	0,57(0,32)
Kupang City	7	0,05(0,00)	0	0,00(0,00)	2	5,05(1,00)	9	0,05(0,00)
Kupang Rural	2	0,45(0,65)	2	0,28(0,05)	0	0,00(0,00)	4	0,28(0,35)
Sub-total	16	0,09(0,18)	8	3,38(4,34)	38	0,62(0,59)	62	0,57(0,93)
All respondents								
Yogyakarta City	10	0,21(0,25)	18	1,89(0,25)	19	0,95(2,37)	47	1,42(2,37)
Bantul	3	1,06(3,86)	15	2,13(3,86)	57	0,53(0,80)	75	0,53(0,80)
Denpasar City	3	1,10(1,36)	6	3,40(5,51)	58	0,73(0,92)	67	0,73(0,92)
Buleleng	12	0,40(0,36)	1	3,64(0,00)	91	0,57(0,16)	104	0,57(0,16)
Kupang City	11	0,05(0,00)	3	3,52(3,92)	2	5,05(0,10)	16	0,05(0,10)
Kupang Rural	6	0,20(0,73)	5	1,40(1,88)	7	1,04(1,27)	18	1,04(1,27)
Total	45	0,21(0,52)	48	2,48(4,27)	234	0,61(0,79)	327	0,65(0,79)

## Discussion

In this study, we assess the accessibility of healthcare facilities for individuals with disabilities in Indonesia through primary data collection, investigating the factors associated with health-seeking behaviors and national insurance scheme utilization, with sufficient sample representation at the district level. Decisions regarding health service utilization, whether through the JKN or OOP payments, varied by location and district. Being diagnosed by a healthcare worker is associated with two outcomes, with those attending hospitals more likely to utilize their JKN card owing to high costs. Respondents who opted not to access healthcare services preferred to purchase over-the-counter medications or were reluctant to wait in line to consult with healthcare professionals.

Our results indicate that nearly all respondents experienced healthcare needs and sought assistance when necessary. The majority of the respondents visited healthcare facilities, including PHCs, hospitals, and independent general practitioner/midwife practices. Although this health needs percentage rate is slightly higher than the results of previous studies conducted in Peru (85%), Bangladesh (85%), and Indonesia (86%), we believe that the results are consistent [14, 30, 31]. Consistent with other studies, we have also limited the time constraint to 1 year. However, our findings confirm that individuals with disabilities have higher healthcare needs, making them a vulnerable group in the healthcare system, requiring attention in the national health priority agenda [16].

Having the national health insurance facilitates better decisions of individuals with disabilities to access formal healthcare facilities. Our finding of the JKN coverage percentage is similar to a prior report of the coverage of the entire population in the Indonesian monitoring system of UHC progress [12, 17, 32]. Nearly all respondents registered in the JKN program continue to mark a significant milestone in progress in covering all people with disabilities, since 2021, when 31% of PWDs were not registered. Although overall JKN utilizations among persons with disabilities appears modest, those registered participants were most likely to access the health services compared to PWDs who were not registered [33]. Those accessing PHCs and hospitals automatically use the JKN to cover their health bills. Furthermore, most of those who decided to purchase over-the-counter medicine or consume traditional medicines reported purely preferring those decisions without any perceptions of poor health service quality [24]. Notably, being covered by the JKN is associated with better decisions to access formal healthcare facilities. While further actions are required to accelerate the translation of high enrollment into effective, disability-inclusive health service utilization, the Indonesian government’s efforts to register PWDs in the JKN system represent an important step toward achieving disability inclusion in UHC.

The affordability of medical service costs differs across facilities and regions. Although fewer than 20% of our respondents failed to provide data, we still consider it to give us a hint regarding the OOP expenses encountered by PWDs in the six districts. Generally, the costs for medical services are manageable, except for hospital expenses, which have become increasingly unaffordable in Denpasar, Buleleng, and Kupang City. This increase in expenses is attributed to increased regional incomes and higher living costs. This situation is concerning, as most PWDs are unemployed and lack personal income. Particularly in the Kupang Rural district, hospital access can be costly owing to limited availability, as several residents have low income and depend on the JKN and health centers. In contrast, respondents living in Special Region of Yogyakarta (Yogyakarta City and Bantul) benefit from a special local insurance program for health services not covered by the JKN. Although the central government has urged local governments to eliminate all forms of regional guarantee funding, the Yogyakarta government has retained its budgeting scheme [28]. Disparity is inherent in a vast healthcare system, such as Indonesia, and the capacity of local governments to analyze and formulate local policies is crucial as they have a deeper understanding of their communities and local contexts [22].

Moreover, variations in the study area influence PWDs’ decisions to seek health services and access free health services. This measure, however, is limited, as it equates barriers with the proportion of JKN non-use and does not account for other structural, financial, or social constraints affecting PWD access. Compared with those residing in other study locations, respondents living in the Kupang Rural and Kupang City districts tend to utilize their JKN when accessing health services. Additionally, the percentage of visits to formal health facilities in both areas is lower. Limited income, along with having a disability, restricts access to free healthcare or results in having none at all [34, 35]. Conversely, of note, the percentage of PWDs who access health services and reside in rural areas, including Bantul and Buleleng districts, is lower than that of those living in urban areas. Ultimately, limited income combined with rurality significantly hinders access to healthcare services for PWDs, and it worsens when their desired health services are not available and affordable [36].

The perception that the cost of the service will be higher influences the utilization of the JKN or the decision not to seek treatment at health facilities. Some PWDs still believed to experience difficulties in accessing health facilities owing to barriers associated with accessibility and affordability. In this study, perceptions of PWDs regarding their ability to pay for services and to reach healthcare facilities are used to illustrate the dimensions of accessibility and affordability, with these indicators derived from the reasons they reported for not accessing healthcare [23]. One in 10 PWDs opted not to access health services in the six regions because they believed they lacked the financial capacity or perceived it as challenging to reach health facilities owing to their disabilities. Although the issue of affordability of the health services may be addressed by the JKN, transportation costs for this group are frequently perceived as prohibitively high for those facing economic constraints. These findings support recommendations from a prior study that, to overcome financial barriers beyond direct medical costs, the government should provide concessions to cover indirect expenses, such as transportation [21]. In parallel, the Government of Indonesia and relevant stakeholders should ensure that PWDs and their families are aware of the social assistance schemes designed to improve both affordability and access to healthcare.

The severity of the health problems encountered by PWDs is impacted by the decision to access health facilities or simply purchase the medicine. Those who opted to buy medicines or seek traditional medicines are individuals with health issues that are less complex than those who access public health services [37]. Findings from other studies pertaining to decisions to self-medicate in Chile and Vietnam support this assumption [38, 39]. Users of public healthcare facilities tend to consult with clinical healthcare workers for life-threatening conditions, including cardiovascular diseases, sexually transmitted infections, and gynecological disorders. Our findings further reinforce this assumption, emphasizing the association between having been diagnosed by a healthcare worker and the decision to utilize health services and engage with the JKN. To ensure that the health status of PWDs and the use of JKN or the purchase of medicine are appropriate, we recommend that the Indonesian government enhance its response to the health needs of PWDs by developing scheduled home visit services. This program can improve the quality of life for PWDs and mitigate the high costs of treatments that require additional health services.

Moreover, preferences in self-medication decisions lead us to question the appropriateness of the medications acquired. More than 60% of our respondents stated that they did not access health facilities because they were already confident in the effectiveness of the medicine. This finding would not pose an issue when the problem is mild and can be resolved using the self-medication practice [40]. Without a proper diagnosis from a physician, we are left wondering about the necessity of the medication, particularly as the disability condition may present more symptoms, suggesting that they could require more complex treatment than individuals without disabilities. It is likely that their focus is more on alleviating the accompanying symptoms, although the most concerning issues can arise. We have suggested further research associated with our uncertainties by conducting a survey of PWDs who visit pharmacies solely to purchase medicine, followed by a detailed examination of their health conditions by healthcare professionals to verify the accuracy of self-medication among PWDs.

The association between health service-seeking patterns at health facilities and marital status, as well as the hard-of-hearing requires further investigation. A systematic review stated that individuals who are hard of hearing are more likely to visit healthcare facilities; however, they experience substantial challenges in communicating with healthcare workers, who are not trained to communicate with them [41]. Although prior studies have suggested that individuals with a disability tend to receive support from family when seeking health care, and the large number of family members of PWDS can increase income and contribute to better individual access to healthcare, no evidence has shown the link between having a partner and the decision to seek help [31, 42, 43]. Further studies are needed to investigate how the encouragement from PWD spouses can differ across various disability groups and why it is significant.

### Strengths and weaknesses

With direct surveys of the disability group using a household approach, the strengths of our method should be highlighted. In Indonesia, this study has successfully conducted a multiple analysis of the patterns of health service-seeking behaviors at formal health facilities and JKN utilization to cover service costs for general health services [33]. Our survey data represent the PWD populations based on the type of disability across the six regions. During the data collection process, quality assurance was applied to the questionnaire and the outcomes of the enumerator interviews. Another strength of this study was that it included data from three research sites, allowing the findings to represent district-level variations in healthcare access and JKN utilization among PWDs. By capturing data from multiple districts, the study provided a broader, more generalizable view of the factors affecting insurance enrolment and service use, which can inform local and regional policy planning.

This study had several limitations. First, we have tried but failed to record family expenditure and income data because most of our respondents could not recall their expenses. Second, although our data successfully captured the decision to use the JKN at each healthcare facility, we were unable to note the frequency of need at each location. The data we present reflects the most recent visit. Third, data on persons with disabilities obtained from Social Services Offices across study sites may already have been reached by government programs and supported in registering for the JKN system, and may therefore be biased toward this group. Consequently, persons with disabilities who remain unengaged with health and social service systems may be underrepresented, which could lead to an overestimation of coverage and access. Fourth, the results may be affected by recall bias, as respondents were required to rely on self-reported information, and if respondents failed to recall certain information, it resulted in missing data. For example, out-of-pocket (OOP) expenditures—which were not addressed analytically—potentially affect the interpretation of the affordability of medical care. Fifth, this study did not examine variables related to patient experience, perceived quality of care before accessing health facilities, or the use of the JKN card, all of which may be important factors influencing health care–seeking behaviours. Sixth, this study did not consider the diverse needs and challenges faced by individuals with sensory or physical disabilities, nor did it take into account different levels of severity in health needs. Seventh, this study is limited in that it relies primarily on descriptive percentages, equates barriers with JKN non-use without considering other structural, financial, or social constraints, and does not examine in depth the structural and developmental differences across regional systems that may influence the decisions of PWDs to enrol in insurance and seek care. As local health systems function as self-organising systems—managing resources and responding to challenges autonomously—further study of the dynamics emerging within each local system is needed to better understand them and guide improvement programmes at both regional and central levels. Lastly, although data on the inclusiveness of health services are crucial, we opted not to include them to maintain the consistency and clarity of our arguments. These findings will be presented in our other study to demonstrate the comprehensiveness of the assessment using inclusivity indicators for health facility readiness, such as the provision of inclusive infrastructure for individuals using wheelchairs, white canes for visually impaired individuals, staff trained for inclusive communications and loudspeakers and signs for those who are hard of hearing [44].

## Conclusion

Although most of the people with physical and sensory disabilities are registered in the Indonesian national health insurance, only half of them decided to access healthcare services across six districts, reflecting an unfinished UHC agenda with disability inclusion. Barriers related to the ability to pay medical and transportation costs to have a health service and the ability to reach the JKN-designated health facilities still continue to hinder the health service-seeking behaviors of PWDs. The Indonesian government, both national and local, should proactively ensure service accessibility to convert the total number of participants into comprehensive health service coverage for PWDs. This includes regular monitoring of PWDs, particularly those with more severe conditions, who face high direct and indirect costs, have limited ability to use JKN, and encounter other barriers to accessing care.

## Electronic Supplementary Material

Below is the link to the electronic supplementary material.

Supplementary Material 1: Supplementary Figure 1. Distributions of people with disabilities based on their JKN registration status and their decisions to utilise the JKN scheme to cover their medicine bills in the Special Region of Yogyakarta (DIY), Bali and East Nusa Tenggara.

## Data Availability

Due to the presence of individual-level information that could potentially compromise the confidentiality of our respondents, our survey dataset is not publicly accessible. Datasets may be obtained from the corresponding author upon reasonable request.

## Abbreviations

BPJS Kesehatan: Badan Penyelenggara Jaminan Sosial Kesehatan - The National Health Insurance Agency
ICW: the Indonesia Corruption Watch
JKN: Jaminan Kesehatan Nasional – National Health Insurance
NTT: Nusa Tenggara Timur - East Nusa Tenggara
OOP: Out-of-pocket
PBI: Penerima Bantuan Iuran JKN - JKN subsidized participants
PHC: Primary Healthcare Center
PWD: Persons with disability
SUSENAS: Survei Sosial Ekonomi Nasional - National Socioeconomic Survey
UHC: Universal Health Coverage
