# Supplementary figures and images for: Achieving universal health coverage with disability inclusion: a household survey analyzing healthcare access and national health insurance utilization for individuals with disabilities in Indonesia
Source: BMC Health Serv Res. 2026 Apr 16;26:760. doi: 10.1186/s12913-026-14572-5 (PMC13217835; doi:10.1186/s12913-026-14572-5)

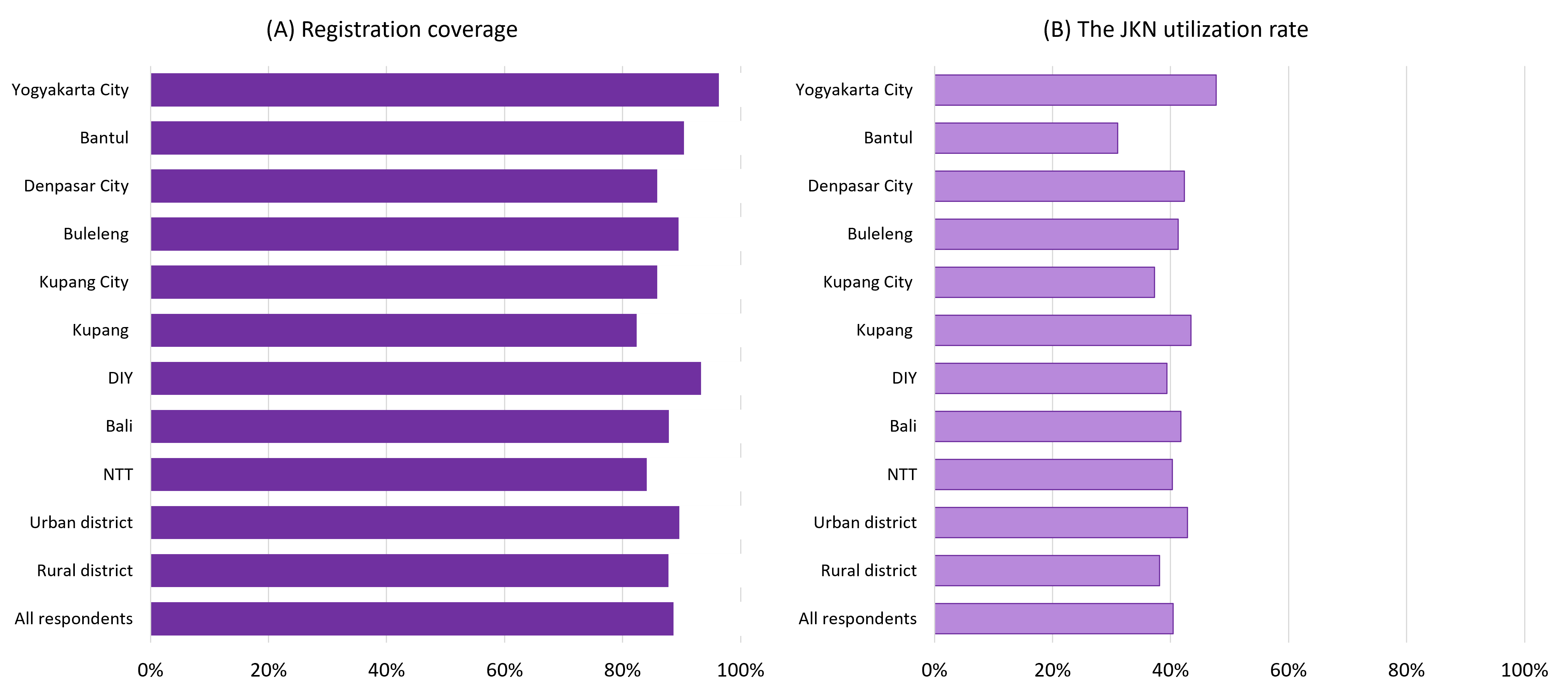

Supplement: Supplementary file 1 — Supplementary Material 1: Supplementary Figure 1. Distributions of people with disabilities based on their JKN registration status and their decisions to utilise the JKN scheme to cover their medicine bills in the Special Region of Yogyakarta (DIY), Bali and East Nusa Tenggara. [file 12913_2026_14572_MOESM1_ESM.tiff]
